# Metabolic profiling reveals nutrient preferences during carbon utilization in *Bacillus* species

**DOI:** 10.1038/s41598-021-03420-7

**Published:** 2021-12-13

**Authors:** James D. Chang, Ellen E. Vaughan, Carmen Gu Liu, Joseph W. Jelinski, Austen L. Terwilliger, Anthony W. Maresso

**Affiliations:** The Department of Molecular Virology and Microbiology, Baylor College of Medicine, Houston, TX USA

**Keywords:** Metabolomics, Microbiology, Bacteriology

## Abstract

The genus *Bacillus* includes species with diverse natural histories, including free-living nonpathogenic heterotrophs such as *B. subtilis* and host-dependent pathogens such as *B. anthracis* (the etiological agent of the disease anthrax) and *B. cereus*, a cause of food poisoning. Although highly similar genotypically, the ecological niches of these three species are mutually exclusive, which raises the untested hypothesis that their metabolism has speciated along a nutritional tract. Here, we developed a pipeline for quantitative total assessment of the use of diverse sources of carbon for general metabolism to better appreciate the “culinary preferences” of three distinct *Bacillus* species, as well as related *Staphylococcus aureus*. We show that each species has widely varying metabolic ability to utilize diverse sources of carbon that correlated to their ecological niches. This approach was applied to the growth and survival of *B. anthracis* in a blood-like environment and find metabolism shifts from sugar to amino acids as the preferred source of energy. Finally, various nutrients in broth and host-like environments are identified that may promote or interfere with bacterial metabolism during infection.

## Introduction

One key hallmark of pathogens is their ability to use hosts’ nutrients for growth and replication^1,2^. Bacterial pathogens, in particular, are thought to have specialized from more environmental isolates in this regard^3^. For example, *B. anthracis*, the causative agent of anthrax, evolved to acquire heme from the oxygen carrier protein hemoglobin^4–6^. Once imported in the bacterial cell, the heme porphyrin ring is broken and the central iron atom liberated^7^. Two plasmids pXO1 and pXO2, which are not observed in other *Bacillus* species, are responsible for the virulence of *B. anthracis*^8,9^. Transformation of these virulence plasmids into certain biovars of *B. cereus* has been demonstrated to result in bacteria that can cause anthrax-like disease^10,11^. Bacterial pathogens have adapted their metabolism to specifically exploit what the host offers in nutrients; heme acquisition is a good example. Competition between the host and pathogens for common resources is the basis of the concept nutritional immunity, which is a biochemical means by which the host controls nutrient levels to keep bacterial growth at bay^12–14^.

*Bacillus anthracis* is the etiological agent of the deadly disease anthrax^15–17^. One of its more defining features is its ability to replicate to very high numbers in mammalian blood and tissues using host-derived nutrients. As such, *B. anthracis* is often used as a model bacterial pathogen for the study of host nutrient uptake during infection^3–5^. Its infectious cycle begins when spores enter the host through an open wound, is inhaled, or is ingested. Spores then germinate inside the host into fully-replicative and growing vegetative cells. This life cycle is in stark contrast to *Bacillus cereus*, a member of *Bacillus cereus sensu lato* group, which usually causes mild symptoms although still capable of causing severe pathologies^18–21^ Another extensively studied *Bacillus* species, *Bacillus subtilis,* is a non-pathogenic soil-dwelling bacteria utilized for food fermentation and the study of bacterial physiology. Both *B. cereus* and *B. subtilis* are known for their extensive genomic variety at the species level. In addition, they are both phylogenetically distinct from highly pathogenic *B. anthracis,* with *B. subtilis* sharing less than 20 percent of the amplified fragment length polymorphism markers with *B. anthracis*^22–24^. All this genomic variations are surprising given that most species in the genus *Bacillus* are normally found in soil as nonpathogenic environmental bacteria. In fact, extreme pathogenicity and virulence of *B. anthracis* is particularly striking when compared to other *Bacillus* species given their shared normal environmental niche, which makes divergence in metabolism at both inter- and intra-species level particularly intriguing^8–11,25^. Whereas it is clear from many studies that secretion systems, secreted toxins, effectors, and adhesins clearly have evolved to assist in host colonization and resistance to host immunity, less clear is what delineates non-pathogenic from pathogenic strains or species when it comes to metabolism of critical nutrients^26–28^. Although there have been previous studies that investigate metabolism of *B. cereus* from the perspective of nutrient utilization, metabolism of *B. anthracis* has been primarily studied from the perspective of metabolic enzymes, a global genomic analysis, or characterization of metabolic regulators^29–35^. In an attempt to define the nutritional preferences of pathogenic and non-pathogenic bacteria, we examined the ability of three species of *Bacillus* (*B. anthracis*, *B. cereus*, and *B. subtilis*), as well as *Staphylococcus aureus*, to utilize 189 distinct sources of carbon in Biolog’s Phenotype Microarray (PM1 and 2) carbon utilization plates designed to emphasize metabolic output arising from differences in metabolic setup. We report the generation of a robust classification scheme that bins metabolic output as a function of chemical class, the use of this system to decipher nutrient preferences between species, how such preferences change in a host-like environment, and the identification of compounds that may poison metabolic networks.

## Results

### Overall trends in metabolic utilization of carbon sources

**Figure 1:**
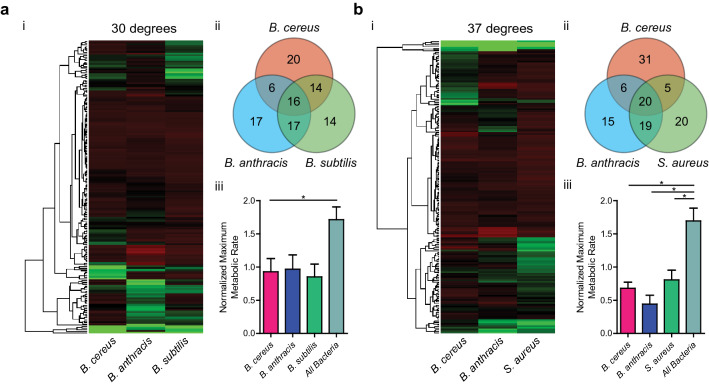
Metabolic rates for carbon sources in bacteria show variations and groupings. (**a** and **b**) Maximum metabolic rates of nutrients for bacteria incubated at 30 °C (**a**) and 37 °C (**b**). (i) Nutrients are hierarchically clustered by their chemical structures (dendrograms, left) and metabolic rates observed are shown as heat maps (right) with each column representing results from different bacteria. (ii) Venn diagrams of nutrients are shown with numbers reflecting the count of nutrients that had metabolic rates statistically greater than the overall average rate (*n* = 3, *p* < 0.05, unpaired Student’s t-test). (iii) Normalized maximum metabolic rates for nutrients well-utilized by one bacteria are compared against nutrients well-utilized by all bacteria. Bars represent averages of all nutrients that had statistically higher metabolic rate than the overall average rate. Error bars represent standard error of the mean. Maximum metabolic rate for each nutrient is an average from three independent experiments (*n* = 3, *: *p* < 0.05, unpaired Student’s t-test). Figure created with R v3.5.3 (https://www.R-project.org) and GraphPad Prism 5 (https://www.graphpad.com).

We sought to determine whether the maximum metabolic rate could be used as a metric to compare bacterial nutrient preferences under different environmental conditions for *Bacillus anthracis* Sterne, *Bacillus cereus* ATCC 10987, *Bacillus subtilis* ATCC 2091, *and Staphylococcus aureus* LAC (Supplementary Fig. S1a). Metabolic activity varied with time for bacteria grown on different carbon sources, indicating the assay had a significant dynamic range (Supplementary Fig. S1b). Data for bacteria incubated at their optimal temperature were used when two different temperatures were tested (Supplementary Fig. S1c). Heat maps were used to aid in the visualization of the data. Examination of metabolic rates showed that while few nutrients were well-utilized in all bacteria, a select group of nutrients were utilized exceptionally well by a given species, a finding effected by temperature (Fig. 1ai, bi). With normalized maximum metabolic rate as the metric, the number of nutrients that gave greater than the overall average rate was counted to show the overlap in utilization of the same nutrient between different species (Fig. 1aii, bii). At 37°, 20 nutrients were utilized at above the average rate among all three bacteria tested, while there were groups of nutrients observed to be better-utilized in one bacteria alone (31 for *B. cereus*, 15 for *B. anthracis*, and 20 for *S. aureus*). Similar distribution was observed for bacteria incubated in 30° as well, although *B. cereus* once again had the greatest number of nutrients that were utilized (20 for *B. cereus,* 17 for *B. anthracis,* and 14 for *B. subtilis*). Six of these nutrients were common to both lists (5-keto-d-gluconic acid, d-arabinose, d-ribose, d-xylose, l-arabinose, and l-lyxose) and all of them were either carbohydrates or derivatives (Supplementary Tables S1 and S2). These nutrients are either pentoses or its’ derivatives, the smallest carbohydrates that can isomerize to cyclic forms and serve as building blocks for oligomers in the cell, which may hint at why they are preferred. When averages of maximum metabolic rates of nutrients that were well-utilized by only one bacteria were compared to that of nutrients well-utilized by all bacteria, it was observed that these nutrients resulted in higher rates as compared to nutrients well utilized by one bacteria at 37° (0.68 for *B. cereus*, 0.44 for *B. anthracis*, 0.80 for *S. aureus*, 1.70 for commonly well-utilized, *n* = 3, *p* < 0.05, unpaired Student’s t-test) (Fig. 1biii). This may indicate that while choices of carbon utilization are distinct for each species, and these differences result in greater rates of metabolism, they also have core parts of metabolism that are common, but generally work at lower rates. It is interesting to also note that *B. anthracis* shared more common nutrients with *B. subtilis* at 30° (Fig. 1aii) and *S. aureus* at 37° (Fig. 1bii) than it did with *B. cereus*, which was unexpected.

### Chemical property preferences during nutrient use

**Figure 2:**
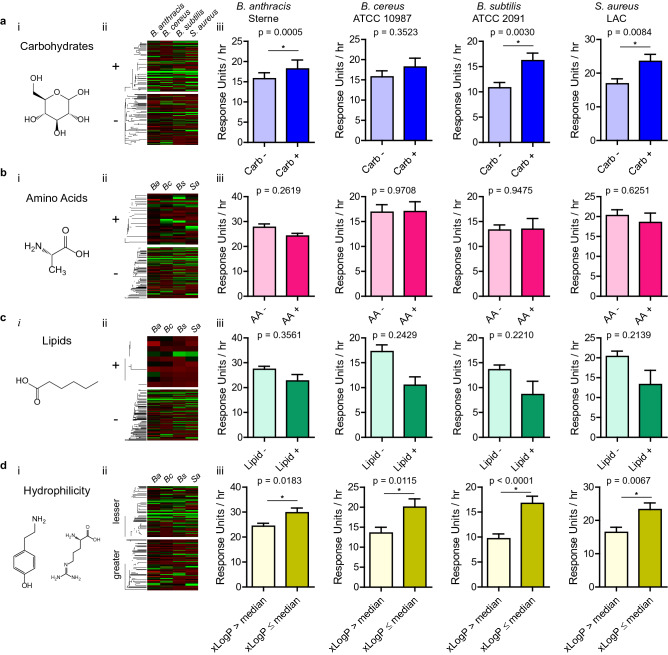
Metabolic rates correspond to certain chemical properties of nutrients. (**a**–**d**) Four chemical properties of nutrients examined with the structure of an example from each category (i): (**a**) carbohydrates (shown: D-glucose), (**b**) amino acids (shown: L-alanine), (**c**) lipids (shown: caproic acid), and (**d**) hydrophilicity as represented by partition coefficient (shown: tyramine and L-arginine). (ii) Heat maps of maximum metabolic rates for nutrients with nutrients in the category for chemical property under question (+ or lesser) or did not (− or greater). Nutrients are hierarchically clustered by their chemical structural similarities using atom-pair distances. *Ba: B. anthracis, Bc: B. cereus, Bs: B. subtilis, Sa: S. aureus.* (iii) Average maximum metabolic rates for nutrients by chemical property (blue: carbohydrates, red: amino acids, green: lipids, yellow: hydrophilicity / partition coefficient). Bars represent averages of all nutrients categorized by chemical property. Error bars represent standard error of the mean. Maximum metabolic rate for each nutrient is an average from three independent experiments (*n* = 3, *: *p* < 0.05, unpaired Student’s t-test). Figure created with R v3.5.3 (https://www.R-project.org) and GraphPad Prism 5 (https://www.graphpad.com).

Nutrients used in this study have a wide variety of chemical properties. This fact can be leveraged to determine the types of nutrients bacteria prefer to eat. We classified nutrients into distinct “food groups” based on their chemical properties as queried through NCBI PubChem: carbohydrates, amino acids, lipids, and hydrophobicity according to their calculated partition coefficient (xLogP3) (Fig. 2ai, bi, ci, di)^36^. Nutrients were hierarchically clustered according to their chemical structural similarities as measured by atom-pair distances using ChemmineR R package within groups^37^. Maximum metabolic rates were standardized to mean of 0 and standard deviation of 1 for each bacteria incubated under their optimal growth temperatures, and visualized as heat maps for comparison, with ‘ + ’ and ‘ − ‘ indicating groups of nutrients that either belonged or not to the “food group,” respectively (Fig. 2aii–dii). The average maximum metabolic rate for carbohydrates was greater than that of non-carbohydrates for *B. anthracis* (31.32 for carbohydrates, 23.46 for non-carbohydrates, *p* = 0.0005), *B. subtilis* (16.19 for carbohydrates, 10.80 for non-carbohydrates, *p* = 0.0030), and *S. aureus* (23.52 for carbohydrates, 16.84 for non-carbohydrates, *p* = 0.0084, all unpaired Student’s t-test) (Fig. 2aiii). This stands in contrast to amino acids and lipids, where no statistically significant differences were observed between nutrients categorized under these properties (Fig. 2biii, ciii). As for hydrophobicity, the median value of xLogP for all nutrients, − 2.3, was used as the dividing point, with xLogP less than or equal to the median as being deemed relatively hydrophilic and greater as hydrophobic. All four species of bacteria incubated under their optimal temperature had average raw maximum metabolic rates for hydrophilic nutrients greater than hydrophobic nutrients (Fig. 2diii). These results highlight facile metabolic utilization of carbohydrates, as opposed to amino acids and lipids, when bacteria are constrained to primarily one nutrient as their carbon source. Superior utilization of hydrophilic nutrients is also suggestive of carbohydrate metabolism, as 76% of hydrophilic molecules (68 out of 89) are carbohydrates, as opposed to 29% (30 out of 102) for hydrophobic nutrients.

To show that metabolic preferences of bacteria by chemical properties of nutrients were not specific to strains tested, we performed identical metabolic screen of nutrients for four additional strains: *B. anthracis* ANR-1, *B. cereus* ATCC 14579, *B. subtilis* ATCC 6633, and *S. aureus* RN4220. *B. anthracis* ANR-1, a derivative of virulent Ames strain which lacks virulence plasmid pXO2, was chosen to represent more virulent variant of *B. anthracis*. In contrast, *S. aureus* RN4220, a well-characterized laboratory strain, was chosen to represent less pathogenic variant as compared to our initial strain LAC, a CA-MRSA strain known for its ability to cause medically complicated infections. *B. cereus* ATCC 14579 and *B. subtilis* ATCC 6633 were chosen as they represent strains that are genetically distant from initial strains examined. Metabolic endpoints for nutrients classified as carbohydrates were greater than those for non-carbohydrates in same three species as our initial experiment (*B. anthracis*, *B. subtilis*, and *S. aureus*) (*n* = 3, unpaired Student’s t-test) (Supplementary Fig. S4a). There were two differences in this new experiment and the initial experiment: 1. Statistically significant increase of metabolism for *B. cereus* in amino acids was observed, whereas no such increase was seen for all bacteria in the initial four strains, and 2. Increase of metabolism in hydrophilic molecules across all four species was still observed in this new experiment, but only in *B. subtilis* was this increase statistically significant (Supplementary Fig. S4b, d).

### Individual nutrients and their metabolic pathway associations

**Figure 3:**
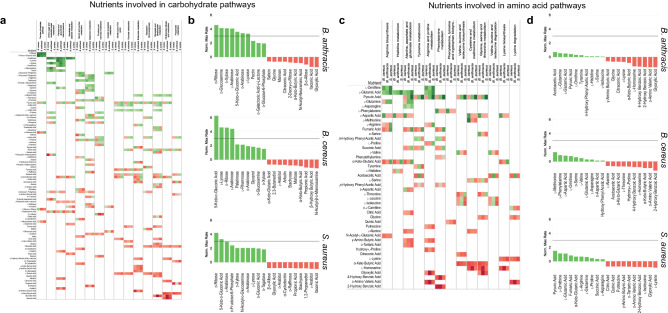
Nutrients are utilized in different pathways with wide range of metabolic rates. (**a** and **c**) Heat map showing normalized maximum metabolic rates for all nutrients associated with carbohydrate pathways (**a**) and amino acid pathways (**c**) (green: higher rates, red: lower rates). For every nutrient (left column), normalized maximum metabolic rates are shown in three columns (*B. cereus, B. anthracis,* and *S. aureus*) for all pathways that the nutrient is associated with. Nutrients are ordered from top to bottom by their overall average metabolic rate. Pathways are ordered from left to right by their average metabolic rate. (**b** and **d**) Bar graphs of normalized maximum metabolic rates for nutrients with top and bottom 10 metabolic rates involved in carbohydrate pathways (**b**) and amino acid pathways (**d**). For each bacteria, maximum metabolic rates for nutrients with 10 highest metabolic rates are shown in green, and 10 lowest metabolic rates are red. Maximum metabolic rates are normalized to average of 0 and standard deviation of 1. Gray lines indicate normalized rate of threshold of 3, which is equivalent to three standard deviations greater than the mean. Each nutrient’s maximum metabolic rate is an average from three independent experiments (*n* = 3). Figure created with Tableau v2020.4.2 (https://www.tableau.com).

Analysis of overall averages of metabolic rates suggests that there exist variations in metabolism at the level of individual nutrients. Using metabolic pathway assignments made for nutrients through KEGG database, individual nutrients and pathways were ordered by their standardized maximum metabolic rate at 37° and laid out as heat maps for carbohydrate (Fig. 3a) and amino acid pathways (Fig. 3c)^38^. There was a large range of metabolic rates even among nutrients utilized by common pathways, hinting that the rate of nutrient utilization is dependent upon specific point at which each individual nutrient enters overall metabolism. There are universally well-utilized nutrients within carbohydrate pathways, such as L-arabinose (4.390 for *B. cereus*, 4.051 for *B. anthracis*, and 2.926 for *S. aureus*), which was consistently involved in the top three out of four pathways (amino sugar and nucleotide metabolism, pentose and glucuronate interconversion, and pentose phosphate pathway). In contrast, analysis of amino acid pathways reflects a more modest degree of utilization and does not show the heterogeneity as observed in carbohydrate pathways. This is more evident when the top and bottom ten nutrients in metabolic maximum rates are separately visualized for carbohydrate metabolism (Fig. 3b) and amino acid metabolism (Fig. 3d). *B. cereus* and *S. aureus* had a small group of nutrients metabolized exceptionally well even within the top ten (four nutrients with normalized rates greater than 3, which is equivalent to three-fold greater rates than the standard deviation, for *B. cereus –* 5-keto-d-gluconic acid, l-lyxose, d-ribose, and l-arabinose; and two nutrients for *S. aureus* – d-ribose and 5-keto-d-gluconic acid), while *B. anthracis* had seven nutrients with rates that exceeded the threshold rate of 3 (d-ribose, d-glucosamine, d-xylose, l-arabinose, 5-keto-d-gluconic acid, d-arabinose, and l-lyxose). In contrast, none of the nutrients involved in amino acid pathways exceeded the threshold of 3. When averages of normalized metabolic rates for carbohydrates is divided by averages of rates for amino acids, the value is three times higher for *B. anthracis* compared to *S. aureus*, and five times higher compared to, which suggests better metabolic utilization of carbohydrates for *B. anthracis* relative to amino acids under these cultured conditions (*B. anthracis*: 10.77, *B. cereus*: 1.86, *S. aureus*: 3.26).

### Nutritional preferences of *B. anthracis* in serum

**Figure 4:**
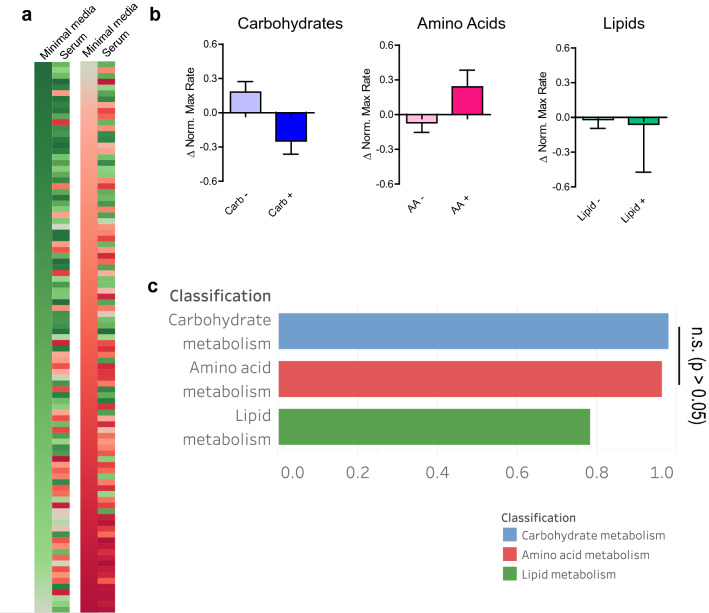
*B. anthracis* has metabolic profile dependent on nutrient availability. (**a**) Comparison of ordered lists of maximum metabolic rates between nutrient-restricted (minimal media) and enriched (serum) environments. Color gradient shows rank of nutrients by their metabolic rate, with green and red representing high and low rates. The ordered list from nutrient restricted condition (left) is shown ordered, and corresponding rank from nutrient enriched condition (right) is placed side as comparison. (**b**) Differences of average metabolic rates between nutrient-restricted and enriched conditions by nutrient category. Bar graphs show differences between average maximum metabolic rates for nutrients by their categories (blue: carbohydrates, red: amino acids, green: lipids). Error bars represent standard error of the mean (*n* = 3, unpaired Student’s t-test). (**c**) Differences of average metabolic rates from nutrients by pathway classification. For each class of pathways, differences in maximum metabolic rates of all nutrients associated with those pathways between nutrient-restricted and enriched conditions were averaged and shown as a bar graph. Colors represent pathway classifications (blue: carbohydrates, red: amino acids, green: lipids) (*: *p* < 0.05, one-way ANOVA) Figure created with GraphPad Prism 5 (https://www.graphpad.com) and Tableau v2020.4.2 (https://www.tableau.com).

Having established the global nutrient requirements for *B. anthracis* under culture conditions, we next sought to compare these results to conditions designed to simulate growth in a mammalian host. As such, carbon sources from the screen were supplemented with 40% fetal bovine serum (FBS) and the entire analysis repeated. Nutrients were ordered according to their maximum metabolic rates (Supplementary Table S3). Interestingly, nutrients well-used in serum by *B. anthracis* were not identical to those well-used in minimal media, with Spearman’s ρ of 0.5050 (most evident in a comparison of heat maps, Fig. 4a). When nutrients are categorized by carbohydrate and amino acid utilization, there is a rather substantial realignment of preferences (carbohydrates: − 0.2474 vs. non-carbohydrates: 0.1809, amino acids: 0.2397 versus non-amino acids: − 0.0700, lipids: − 0.0588 versus non-lipids: − 0.0187) (Fig. 4b). The most striking observation is decrease in utilization of carbohydrates by *B. anthracis* in serum, with corresponding increase into utilization of amino acids as seen by changes to metabolic endpoints (carbohydrates: − 0.2474, amino acids: 0.2397, *p* = 0.0264, unpaired Student’s t-test). This change of metabolism is most apparent when comparing the number of nutrients that have higher normalized metabolic rates in media as opposed to those in serum (for carbohydrates: 50 in media vs. 39 in serum, for amino acids: 11 in media vs. 19 in serum). These metabolic differences in serum are most readily seen when nutrients are categorized by pathway. Analysis of these pathways at the nutrient level for carbohydrates and amino acids (Supplementary Fig. S5) show that decreases in metabolic rates for carbohydrates from media to serum are greatest for certain pentose (d-xylose: − 3.808, d-arabinose: − 3.616, d-ribose: − 4.243) and hexose derivatives (d-galactonic acid-γ-lactone: − 2.897, d-glucosamine: − 4.053). This demonstrates that these simpler carbohydrates, while well-utilized under nutrient-poor conditions in PM environment, are either no longer efficient or preferred as carbon sources in an environment rich with diverse nutrients. Correspondent with changing metabolic utilization preferences, specific preferences for carbohydrate metabolism associated nutrients were no longer observed (Fig. 4c). These results suggest that there are changes to carbon metabolism dependent on the local environment bacteria exist, with *B. anthracis* switching metabolism to favor catabolism of amino acids in serum.

### Metabolic activity of *B. anthracis* in combinations of nutrients

**Figure 5:**
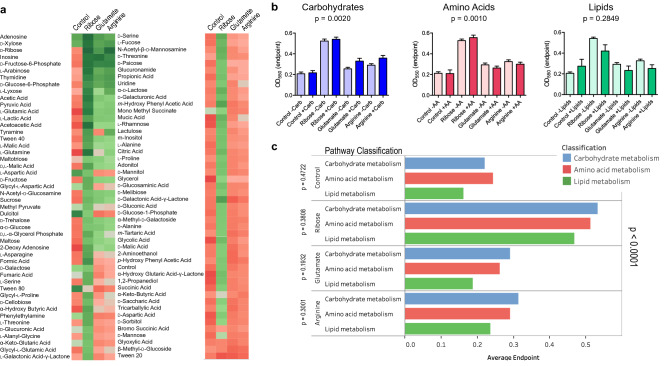
*B. anthracis* metabolism responds differently to specific combination of nutrients. (**a**) Metabolic endpoints of *B. anthracis* in PM1 screen shown as a heatmap. Metabolic endpoints are shown in a gradient from red (low) to green (high) (*n* = 3). Nutrients are ordered by the average metabolic endpoints for all combinations tested. (**b** and **c**) Metabolic endpoints for classification of metabolic pathways by combinations of screen and additional nutrients at concentration of 50 μM. (**b**) groups metabolic endpoints of nutrients by their metabolic pathways and the presence or lack of additional nutrients, while (**c**) shows the overall metabolic endpoints averages for all combinations with additional nutrients (*n* = 3, one-way ANOVA). Figure created with GraphPad Prism 5 (https://www.graphpad.com) and Tableau v2020.4.2 (https://www.tableau.com).

To investigate the possibility of synergy or antagonism of metabolic activity by two different nutrients, we measured metabolic activity of *B. anthracis* incubated on PM1 screens with d-ribose, l-glutamic acid, or l-arginine added to screen of nutrients. These three nutrients were chosen for their utilization efficiency by *B. anthracis* in PM1 screen, as well as their metabolic roles identified in KEGG. Final concentration of these added nutrients was selected to maximize metabolic activity change as seen in previous experiments. Averaged metabolic endpoints are shown as heatmaps with metabolic endpoints shown in a gradient with lowest metabolic endpoints as red and highest with green (*n* = 3) (Fig. 5a). Reflective of results from screens of single nutrients, overall metabolic endpoints for PM1 nutrients with d-ribose supplementation was higher than those for glutamic acid and arginine supplemented plates (0.5309 for d-ribose, 0.2877 for l-glutamic acid, and 0.3201 for l-arginine, Fig. 5b). 93 nutrients with d-ribose supplementation had endpoints higher than the mean endpoints of all nutrients without supplements (mean of 0.2112); in contrast, the number of nutrients that had higher endpoints with supplementation by l-glutamic acid was 51 and l-arginine was 74. This general increase in metabolic endpoints by supplementing nutrient screen with d-ribose was observed in all three categories of nutrients, while for both l-glutamic acid and l-arginine opposite was observed with decreases in endpoints for screen nutrients categorized as amino acids and lipids. Overall, increase in average metabolic endpoints for base nutrients that are carbohydrates and amino acids supplemented with d-ribose was statistically significant, while this was not significant for lipids (one-way ANOVA, carbohydrates: *p* = 0.0020, amino acids: *p* = 0.0010, lipids: *p* = 0.2649). When nutrients were grouped by pathways and their categorical classification, differences in metabolic endpoints for three additional nutrients were not significant. On other hand, there were statistically significant differences between additional nutrients when grouped by categories of base nutrients (Fig. 5c).

## Discussion

Previous studies have shown that pathogens thrive in carbohydrate-rich environments^39^. The results here support this notion. Interestingly, though, there exists a pool of carbohydrates that are well-utilized by all species examined (Supplementary Table S2) regardless of temperature (13 out of 16 for 30°, 15 out of 20 for 37°). It can thus be inferred that most commonalities in these species related to metabolism are the result of the catabolism of carbohydrates. In addition, two pathogens that thrive on mammalian hosts at 37°, *B. anthracis* and *S. aureus*¸ have the most number of well-utilized nutrients in common, reflecting how both bacteria have adapted their metabolism to the temperature of their host. Although large metabolic variations exist at both species and strain levels for all bacteria in the study, common use of the same carbohydrates by the four species surveyed may indicate that it may be challenging to design pathogen-specific antibiotics that selectively inhibit carbohydrate processing pathways in distinct species. More specifically, this commonality raises the question as to why pentoses are utilized better than other carbohydrates in *B. anthracis*, a pathogen noted for its virulence among four species in this study. High metabolic utilization observed in nutrients involved in pentose phosphate pathway (PPP) might offer an explanation. In addition to being catabolized for energy production, these pentoses can also be readily funneled into anabolic pathways through the PPP^40^. Due to their relative chemical simplicity and more basal level of metabolic pathways in contrast to hexoses, metabolic pathways involving pentoses may be functional in the background at all times. Thus providing pentoses to *B. anthracis* may funnel these nutrients to pathways that are ready to newly create biomolecules. This hypothesis of pathogens investing nutrients to expanding metabolic infrastructure, instead of immediately spending it for energetic payoffs, will need to be further investigated.

We also examined nutrients as organic molecules with distinct chemical properties to investigate what factors make one nutrient superior to another in metabolism. Biolog’s Phenotype MicroArray panels are intended to be used as a screen to identify bacteria, and this goal is accomplished by maximizing metabolic output differences between bacteria with corresponding selection of nutrients, which includes some large and complex nutrients that might be challenging for most bacteria to metabolize. Nonetheless, our study shows that remaining nutrients in screens gave us a representative understanding of how different chemical classes of nutrients are utilized among bacteria. Grouping nutrients among their chemical classification led us to conclude some properties of carbohydrates as being favorable for metabolism, and examining chemical structures of various categories of nutrients yields some insights. Variations in carbohydrates arise from different configuration of anomeric carbon atoms and the resulting stereochemistry, which retains overall chemical bond connectivity with same number of carbon atoms. There are only eight possible stereoisomers for aldopentoses, all of which are metabolized into pyruvates. This may explain the higher metabolic utilization of pentoses across all bacteria surveyed here. The relative simplicity of carbohydrates as molecules allow bacteria to metabolize a variety of them with fewer enzymes as compared to other classes of nutrients. A study in *Bacillus thuringiensis* has previously shown that the PPP is preferentially used for carbon metabolism during production of toxin^41^. Similarly, various pathogens in *Streptococcus* tune up expression of virulence factors and carbohydrate utilization enzymes simultaneously^43,44^. Our observation of metabolic utilization in *B. anthracis* and *S. aureus* matches this shift in nutrient preference towards carbohydrates among pathogenic bacteria compared to *B. subtilis.*

The connection between nutrition and bacterial infection has been primarily approached from perspective of host malnutrition and resulting immune dysfunction, as it has been assumed that bacteria are indiscriminate in utilizing all categories of nutrients^44^. Our experiment testing metabolism in *B. anthracis* in combination of PM nutrients and specific nutrients demonstrated metabolic proficiency of this pathogen when consuming certain combinations of nutrients. Thus, it is reasonable to expect them to be more proficient in pathogenesis upon encountering an optimal combination of macronutrients that might be physiologically impractical for the host to adjust or sequester. This could explain why host’s nutritional immunity is geared towards key micronutrients instead, such as iron and biotin, as these linchpins of metabolism are necessary during proliferation phase of bacterial pathogenesis^6,45^. Intriguingly, there also is research showing that a similar strategy of nutrient depletion exists against specific pathogens for amino acid tryptophan, an amino acids with more complicated biosynthetic pathway^46–48^. In this study, we observe that *B. anthracis* has heightened metabolic utilization of amino acids in serum-approximating conditions (Fig. 4) while having higher metabolism for d-ribose, a carbohydrate, in a more austere environment with only one other carbon source (Fig. 5). This suggests that accessibility to amino acids is the rate limiting factor in nutrient-rich environments, while carbohydrates limit the metabolic rate in nutrient-poor environments. It has been previously demonstrated that *B. anthracis* proteolyzes host proteins to obtain branched chain amino acids necessary for metabolism^5^. These facts hint that *B. anthracis* may have to forcibly obtain certain amino acids from the host through virulence mechanisms, and it may remain vulnerable to depletion of amino acids by the host through other means.

We observed that a handful of nutrients in the screen were metabolized with maximum rates much lower than the average. However, it was surprising that some of these nutrients also had among the lowest metabolic maximum rate in the enriched condition, for this indicated that metabolism of *B. anthracis* was interfered by these erstwhile nutrients. Among these, there were five nutrients where the maximum rate did not even reach 30% of the negative control (itaconic acid, 2-hydroxy benzoic acid, glyoxylic acid, β-methyl-d-glucoside, capric acid). One possible explanation is that these nutrients themselves directly act as inhibitors of metabolic enzymes. Given that four out of these five nutrients are not normally used metabolites, it can be argued that these molecules may poison specific components of bacterial metabolism as unprocessed mimics of normal metabolites. Indeed, in case of itaconic acid, its ability to inhibit bacterial growth through metabolic interference has been previously demonstrated^49^. The other explanation is that these nutrients have the ability to tune down metabolism through feedback. In case of glyoxylic acid, a key piece in the glyoxylate shunt that synthesizes carbohydrates from other carbon sources, overall metabolism might be disrupted by the unnatural presence of glyoxylic acid that changes the net chemical output of metabolism into molecular production instead of consumption through glyoxylate shunt^51–53^. In a nutritionally diverse environment, *B. anthracis* may become amenable to metabolic interference on many different fronts as bacteria must simultaneously handle diverse classes of nutrients available. Our study offers glimpses into how antagonizing metabolism through metabolite analogues could be utilized as antibacterial agents.

Finally, a pathway analysis of nutrient utilization between different bacteria yielded both commonalities and differences among nutrients. In addition to pentoses that were well-utilized across all bacteria tested, nucleosides also showed generally high metabolic utilization rates (Fig. 3a). All of these nucleosides have in common d-ribose, a pentose, which is readily metabolized through the PPP. Thus for this class of nutrients, a single rule (presence of pentose) serves as a good proxy for high metabolic utilization. However, other metabolites paint a more complex picture of simple chemical units being the determinant of metabolic utilization. It has been previously demonstrated that acetate inhibits growth of *S. aureus* more than lactate, even though the bacteria has pathways for using both of these simple metabolites^53^. Our metabolic panel results show that low metabolic utilization for acetate in *S. aureus* are in good agreement with these studies. Two overarching themes emerge from our examination of nutrient utilization: 1. There are classes of nutrients that are facile for most bacteria to metabolize, and 2. Metabolic utilization of one specific nutrient for one bacterial species depends on specific circumstances. When basic carbon utilization pathways are analyzed, it becomes apparent that both themes are observed: many nutrients are well-utilized by common pathways across all bacteria, while exceptions to this trend exist across all pathways examined. This suggests that these distinct utilization rates arose as a part of the species’ specialization into their ecological niches with specific mixture of nutrients. It also hints that metabolic specializations of pathogenic *Bacillus* may have arose from their interactions with host organisms as pathogens. These specializations hint that there still could be nutrient-like compounds that could be useful for selectively targeting certain pathobionts amongst a myriad of beneficial or non-pathogenic commensal species among the microbiome.

## Material and methods

### Preparation of bacteria for assays

Frozen bacterial stocks of *B. anthracis* (Sterne and ANR-1), *B. cereus* (ATCC 10987 and 14579), *B. subtilis* (ATCC 2091 and 6633), and *S. aureus* (LAC and RN4220) were added to 1 mL of Luria Bertani media at 1% inoculum and incubated in 30 °C or 37 °C overnight with 160 rpm orbital shaking to stationary growth phase (OD_600_ > 1.5) with kanamycin added (50 μg/mL for *B. anthracis* and *B. cereus*). One mL of bacterial culture was washed twice with 1 mL of deionized water after spinning down in Beckman Coulter Centrifuge (Beckman Coulter, Indianapolis, IN, USA. https://www.beckman.com) for 3 min at 17,000xG. Washed cells were diluted in IF-0a inoculating fluid from Biolog (Hayward, CA, USA. https://www.biolog.com) – here referred to as minimal media – to 81% transmittance equivalent (OD_600_ ~ 0.093) as measured by Beckman Coulter DU800 Spectrophotometer (Beckman Coulter, Brea, CA, USA).

### Bacterial metabolic utilization assay

For growth on 96-well Phenotype MicroArray carbon utilization assay plates (Biolog), 880 μL of washed bacterial cells were added to the assay media of following composition: 10 mL of IF-0a inoculating fluid (Biolog), 120 μL of tetrazolium-based Dye Mix F (Biolog), and assay additives for the assay, with deionized water added to total volume of 12 mL^54^. In the experiment examining the combination of nutrients with Phenotype MicroArray nutrients, 5 mM stock solution for three nutrients (d-ribose, l-glutamic acid, and l-arginine) were first prepared, then 10 μL was directly added to individual wells of Phenotype MicroArray PM 1 plates first and the volume of bacterial suspension adjusted to achieve final additional nutrient concentrations of 50 μM. Full list of nutrients, as well as corresponding PubChem identification numbers, can be found on Supplementary Table S4. Well number 7 of the Phenotype MicroArray plate 2 contained gelatin, which due to its heterogeneous composition was excluded from all further analysis. 100 μL of bacterial cells in the assay media were dispensed into each well of Phenotype MicroArray plate. Plates were incubated in OmniLog ID System (Biolog) at 30 °C or 37 °C for 24 h. Measurements of color changes were made every 15 min (Supplementary Fig. S1). Each Phenotype MicroArray plates was repeated three times for biological replicates.

### Extraction of metabolic endpoints and rates

Raw metabolic data was processed with MATLAB R2017b (Mathworks, Natick, MA, USA. https://www.mathworks.com) to obtain metabolic endpoints and maximum metabolic rates for each nutrient. Metabolic endpoint was defined as the net increase of the metabolic value from the baseline to the exponential moving average of metabolic curve (α = 0.25) at the conclusion of experiment^55^. Fifth degree polynomials were fitted to raw metabolic curves using the MATLAB function polyfit. This polynomial was differentiated with MATLAB function diff to derive a function of metabolic rates, then a table of metabolic rates at all time points generated and the maximum value from the metabolic rate table was chosen.

### Hierarchical clustering of nutrients

For hierarchical clustering of by the chemical structure, chemical structures for nutrients in Phenotype MicroArray carbon utilization screen were queried from PubChem Download Service as SDF files. SDF files were converted to atom distance pairs using R v3.5.3 (R Foundation for Statistical Computing, Vienna, Austria. https://www.R-project.org) with ChemmineR v3.34.1 package sdf2ap function, and fpSim function was used to calculate similarities and generate a distance matrix^37^. The distance matrix of chemical structural similarities was used by hierarchical clustering function hclust from R stats package v3.5.1 and visualized with R gplots package v3.0.1.1 heatmap.2 function. For hierarchical clustering by metabolic data, metabolic data was directly used to calculate a set of pairwise distances by MATLAB function pdist. Euclidean distance was used as the distance metric. Pairwise distances between nutrients were converted into a square matrix with MATLAB function squareform. Resulting distance matrix generated was clustered with R function hclust and visualized with heatmap.2^56^.

### Fetal bovine serum-supplementation metabolic assay

880 μL of washed bacteria suspended in IF-0a media were added to 4.8 mL of Gibco Fetal Bovine Serum (Thermo Fisher Scientific, Waltham, MA, USA. https://www.thermofisher.com), 120 μL of Dye Mix F (Biolog), and 6.2 mL of phosphate buffered saline, pH 7.8, for the final fetal bovine serum concentration of 40% v/v. 100 μL of suspension was added to each well of Phenotype MicroArray carbon utilization plates, and plates incubated at static position in 30 °C or 37 °C for 24 h in Synergy HT Multi-detection Microplate Reader (BioTek, Winooski, VT, USA. https://www.biotek.com) with the color change due to metabolic activity measured as 550 nm absorbance readings taken every 15 min.

### Statistical analysis

Unpaired Student’s t-test and one-way ANOVA with Tukey post-hoc test were performed on GraphPad Prism 5 (GraphPad Software, La Jolla, CA, USA. https://www.graphpad.com). Spearman’s rank correlation coefficients were calculated with Excel. All visualization was performed through GraphPad Prism, R, or Tableau v2020.4.2 (Tableau Software, Mountain View, CA, USA. https://www.tableau.com).

## Supplementary Information

Supplementary Information 1.

Supplementary Information 2.

## Data Availability

All metabolic data used in this study can be found in the electronic supplementary material.
