# Supplementary material for: Metabolic profiling reveals nutrient preferences during carbon utilization in Bacillus species
Source: Sci Rep. 2021 Dec 13;11:23917. doi: 10.1038/s41598-021-03420-7 (PMC8669014; doi:10.1038/s41598-021-03420-7)
Supplement: Supplementary file 1 — Supplementary Information 1. [file 41598_2021_3420_MOESM1_ESM.pdf]

Supplementary Figure S1

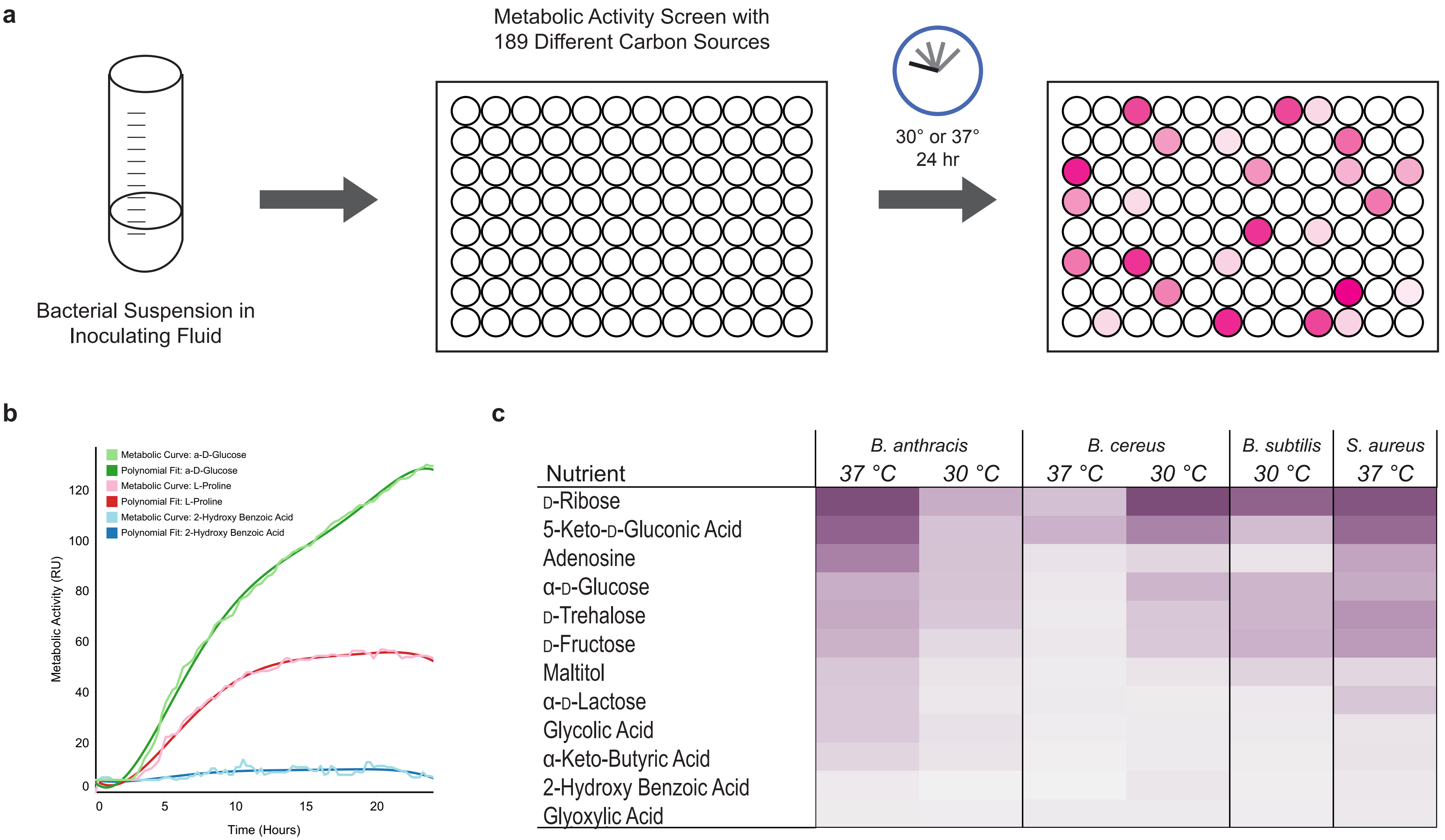

Supplementary Figure S1. Colorimetric assay to monitor metabolic activity in bacteria.

(a) Schematic showing the experimental setup using 96-well plates with nutrients providing a carbon source for bacteria being examined. (b) Examples of raw metabolic data outputs and polynomial fitting for metabolic curves. Metabolic curves over the course of experiment for three nutrients with different degrees of color change are shown: High activity (green) with α-D-glucose, medium activity (red) with L-proline, and low activity (blue) with 2-hydroxy benzoic acid. Light curves show raw metabolic data output as measured by the overall color change, and corresponding dark curves show polynomials fitted to determine metabolic rates. (c) Maximum metabolic rates of bacteria and conditions tested for selected nutrients. Maximum metabolic rates for twelve selected nutrients from the carbon utilization screen are shown to highlight the range of rates measured. Darker shades reflect higher rates, and lighter shades lower rates. Two experiments in separate temperatures (30 degrees and 37 degrees) were performed for *B. anthracis* and *B. cereus* and are shown in two columns. Maximum metabolic rates are averaged from three independent runs. Figure created with Tableau v2020.4.2 (https://www.tableau.com).

Supplementary Figure S2

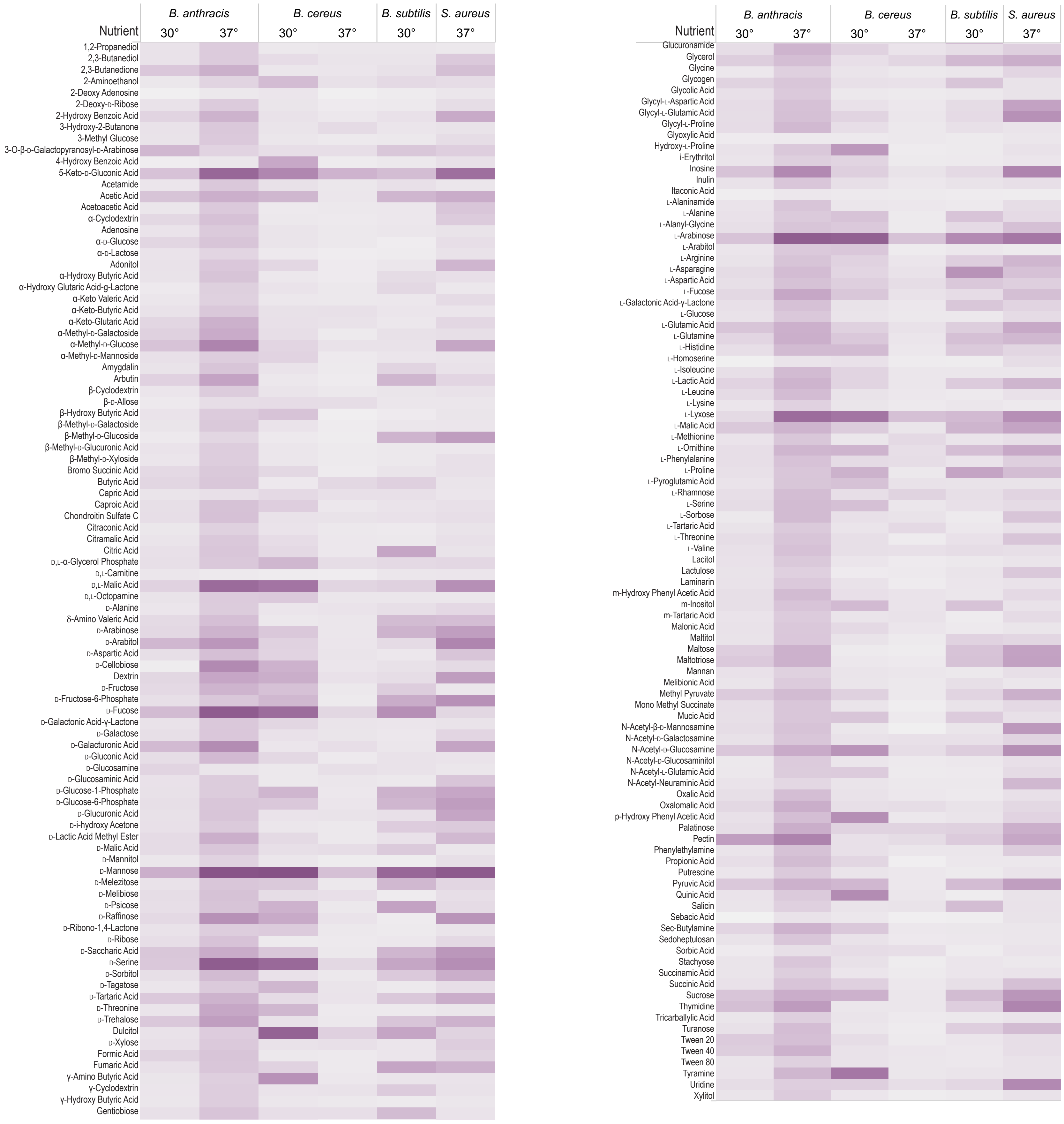

Supplementary Figure S2. Maximum metabolic rates of bacteria in all conditions for all nutrients.

The full list of all nutrients examined in this study is shown with maximum metabolic rates for all bacteria and conditions tested. Darker shades reflect higher rates, and lighter shades lower rates. Figure created with Tableau v2020.4.2 (https://www.tableau.com).

Supplementary Figure S3

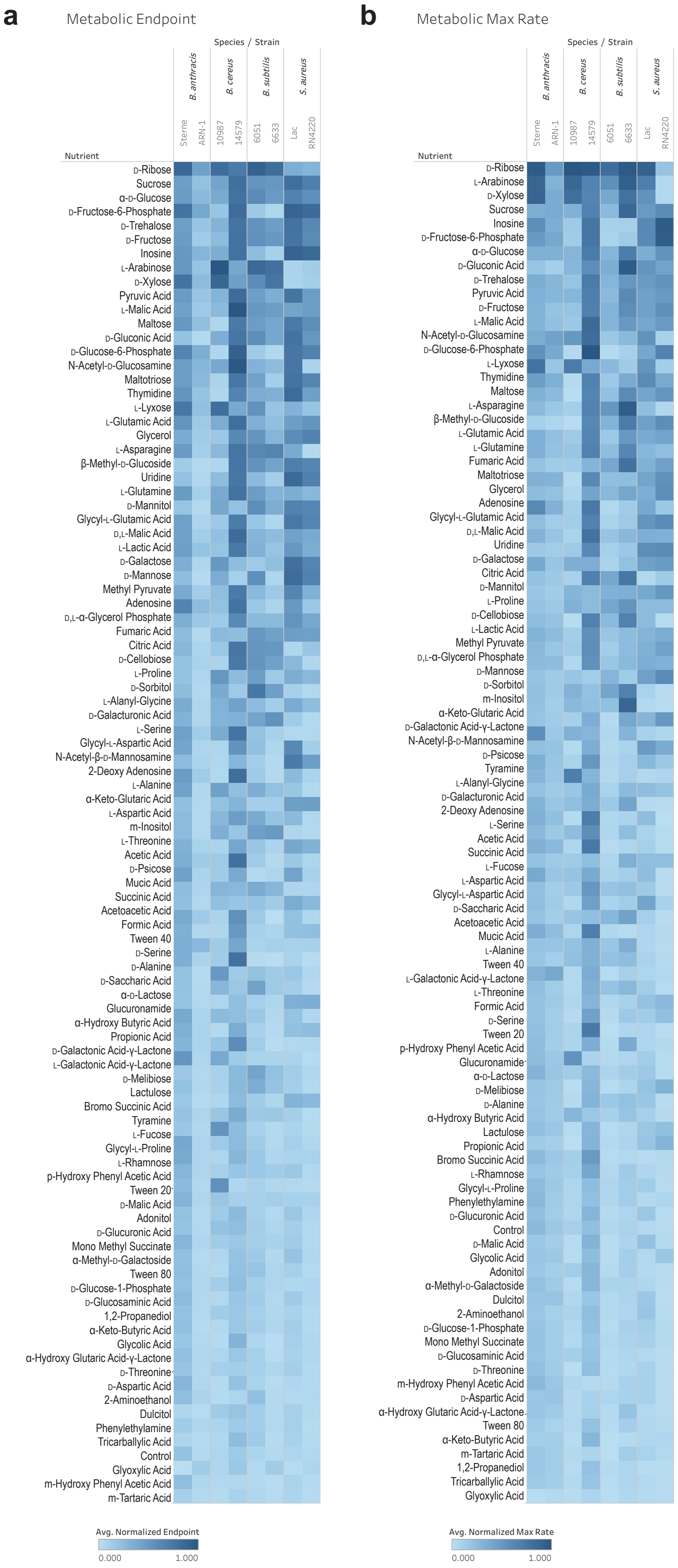

Supplementary Figure S3. Table of metabolic endpoints and maximum metabolic rates for all eight strains of four bacterial species examined.

All measurements are from Phenotype Microarray PM1 screen. Nutrients are ordered from top to bottom in decreasing order of average values for all bacteria. Figure created with Tableau v2020.4.2 (https://www.tableau.com).

Supplementary Figure S4

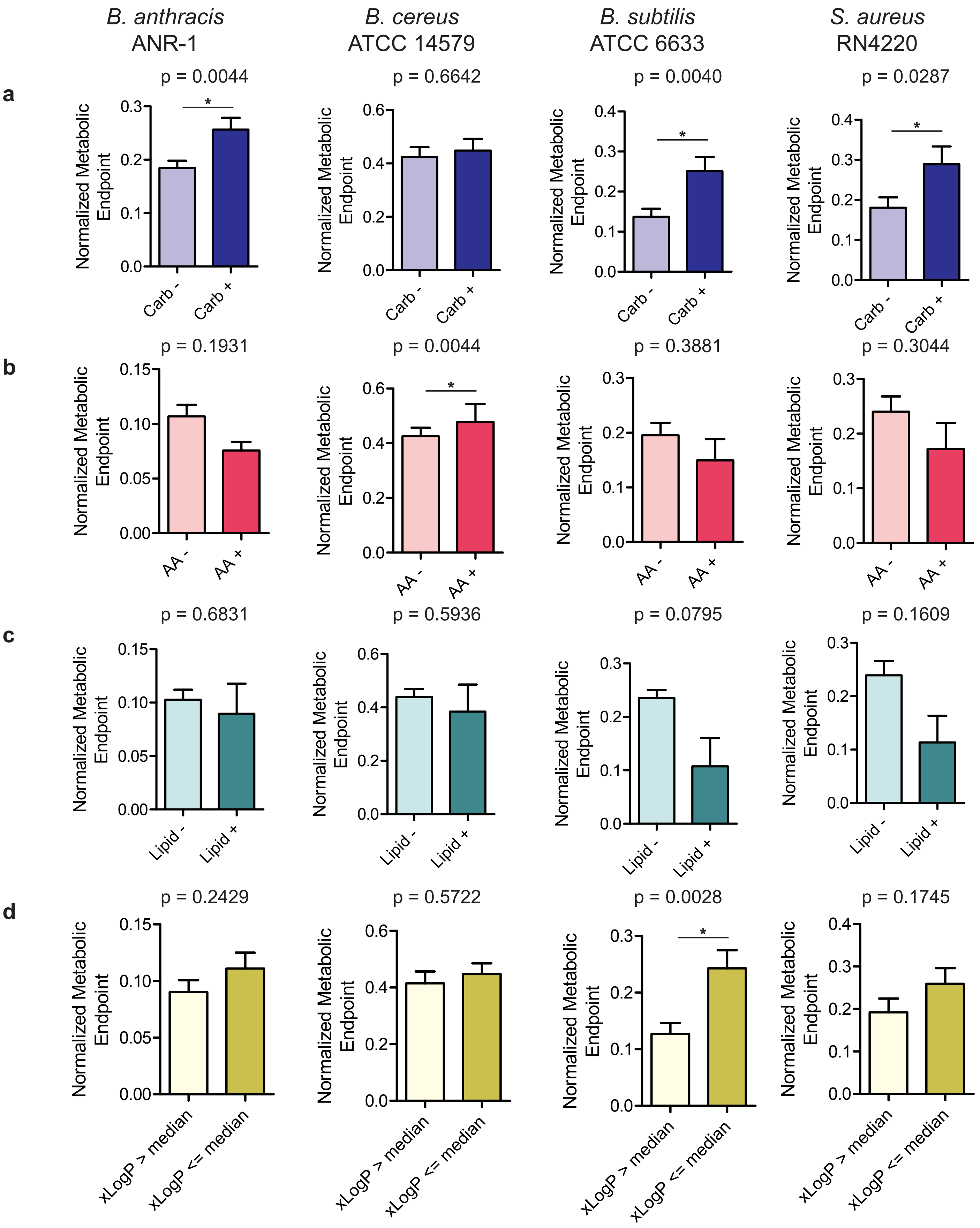

Supplementary Figure S4. Metabolic endpoints for four additional strains screened.

(a-d) Average metabolic endpoints for nutrients by chemical property (blue: carbohydrates, red: amino acids, green: lipids, yellow: hydrophilicity / partition coefficient). Bars represent averages of all nutrients categorized by chemical property. Error bars represent standard error of the mean. Metabolic endpoints for each nutrient is an average from three independent experiments ($n = 3$, *: $p < 0.05$, unpaired Student's t-test). Figure created with GraphPad Prism 5 (https://www.graphpad.com).

Supplementary Figure S5

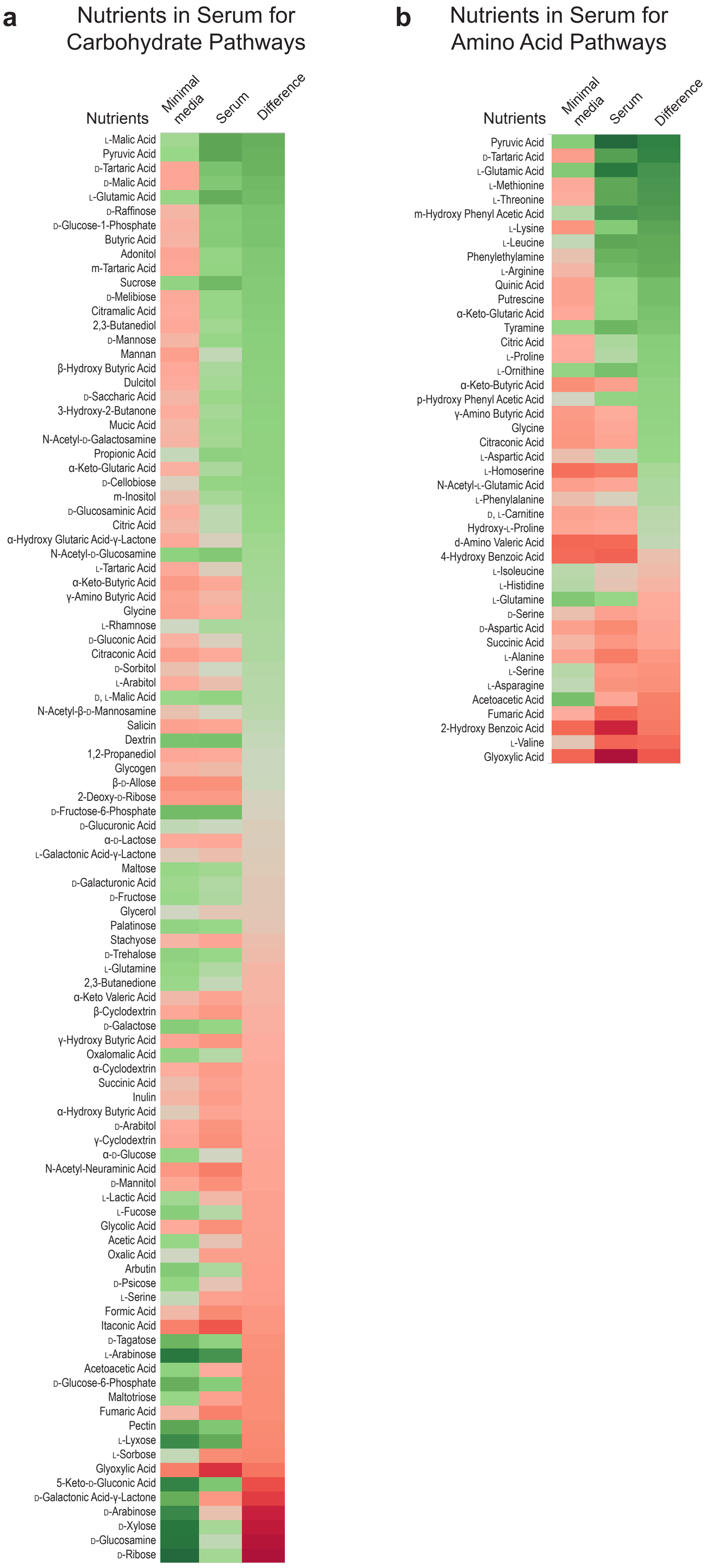

Supplementary Figure S5. Differences in *B. anthracis* metabolic profile between nutrient restricted and enriched environments.

(a and b) Maximum metabolic rates of nutrients associated with carbohydrate pathways (a) and amino acid pathways (b) are shown as heatmaps. Rates from nutrient restricted environment (minimal media, left), nutrient enriched environment (serum, middle), and difference between two (right) are shown. Nutrients associated with more than one pathway are listed in all associated pathways. Figure created with Tableau v2020.4.2 (https://www.tableau.com).
